# A New Era of Antibiotics: The Clinical Potential of Antimicrobial Peptides

**DOI:** 10.3390/ijms21197047

**Published:** 2020-09-24

**Authors:** Katrina Browne, Sudip Chakraborty, Renxun Chen, Mark DP Willcox, David StClair Black, William R Walsh, Naresh Kumar

**Affiliations:** 1 School of Chemistry, University of New South Wales (UNSW) Sydney, Sydney 2052, Australia; k.browne@unsw.edu.au (K.B.); s.chakraborty@unsw.edu.au (S.C.); r.chen@unsw.edu.au (R.C.); 2 School of Optometry and Vision Science, University of New South Wales (UNSW) Sydney, Sydney 2052, Australia; m.willcox@unsw.edu.au; 3 Surgical and Orthopaedic Research Laboratories (SORL), Prince of Wales Clinical School, Prince of Wales Hospital, University of New South Wales (UNSW), Randwick 2031, Australia; w.walsh@unsw.edu.au

**Keywords:** antimicrobial peptides, antibiotic-resistance, antimicrobial activity, peptide-based therapies, cationic peptides, clinical translation

## Abstract

Antimicrobial resistance is a multifaceted crisis, imposing a serious threat to global health. The traditional antibiotic pipeline has been exhausted, prompting research into alternate antimicrobial strategies. Inspired by nature, antimicrobial peptides are rapidly gaining attention for their clinical potential as they present distinct advantages over traditional antibiotics. Antimicrobial peptides are found in all forms of life and demonstrate a pivotal role in the innate immune system. Many antimicrobial peptides are evolutionarily conserved, with limited propensity for resistance. Additionally, chemical modifications to the peptide backbone can be used to improve biological activity and stability and reduce toxicity. This review details the therapeutic potential of peptide-based antimicrobials, as well as the challenges needed to overcome in order for clinical translation. We explore the proposed mechanisms of activity, design of synthetic biomimics, and how this novel class of antimicrobial compound may address the need for effective antibiotics. Finally, we discuss commercially available peptide-based antimicrobials and antimicrobial peptides in clinical trials.

## 1. Introduction

Antimicrobial resistance is not a single grand challenge, but a series of interconnected challenges. In order to prevent an apocalyptic post-antibiotic era, we require the efforts of governments, policy makers, pharmaceutical companies, agricultural workers, healthcare workers, and the general public. The commercialization of antibiotics in twentieth century marked a new era of modern medicine. Today, the number of antibiotic-resistant bacteria continues to rise [1]. While there are many factors that determine antimicrobial-resistance, the global spread of antibiotic-resistant bacteria can be attributed to the misuse of antibiotics and an absence of effective antibiotics released to the market [2]. This crisis requires a global collaboration and comprehensive effort to design and produce effective antimicrobial agents that limit the spread of antimicrobial-resistant pathogens. Currently under extensive clinical research is the naturally occurring class of antimicrobial peptides. This review will explore the benefits and challenges of antimicrobial peptides as therapeutic agents.

## 2. History of Antibiotics and Resistance

**Figure 1:**
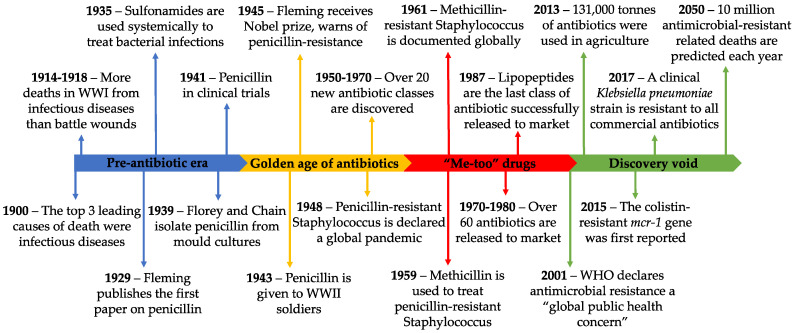
A summary of events in the antibiotic-resistance timeline. WHO, World Health Organization; WWI/II, World War I/II.

Prior to the commercialization of antibiotics, the three leading causes of death were pneumonia and flu, tuberculosis, and gastrointestinal infections [3]. During World War I (WWI), infectious diseases caused more deaths than battle wounds [4]. The serendipitous discovery of penicillin in 1928 marked a new era of modern medicine [5]. Not only did it spark the development of new antibiotics, but it changed the entire drug discovery pipeline. Today, we have a myriad of antibiotics that are effective against a wide range of bacteria. However, the over-reliance on antibiotics came with a cost—a cost we were warned about by Alexander Fleming in his 1945 Nobel prize acceptance speech. He expressed his concern about improper use of penicillin and how easily resistance is acquired with insufficient treatment dosages [6]. It is clear we did not heed this warning and are now suffering the consequences. A brief timeline of antibiotic resistance is provided in Figure 1.

Fleming’s concerns still have far reaching implications. A comprehensive report, aimed to assess rising antimicrobial resistance, has predicted that, by 2050, over 10 million deaths with occur annually as a result of antimicrobial resistant pathogens, culminating to a 100 trillion USD economic burden [1]. Without the use of effective antibiotics, many surgical procedures and treatments that suppress the immune system (such as chemotherapy) will be prohibited [1]. Similar to the impact of viral pandemics, without effective prophylactic treatments, or cures, healthcare systems will be consumed with the spread of uncontrollable diseases.

A variety of factors have led to the progressive increase in antibiotic resistant bacteria; however, they all narrow down to the over-reliance on antibiotics. The use of antibiotics in agriculture and the environment is largely unregulated; only when resistance is widespread are certain antibiotics prohibited [7]. Alarmingly, 80% of all antibiotics are consumed by animals, with the remaining 20% for human use [8]. These powerful antibiotics are used in agriculture as growth promoters and to prevent infection of animals kept in unhygienic environmental conditions. For example, colistin is a last-resort antibiotic for the treatment of unresponsive multidrug-resistant infections. However, its widespread use in agriculture has propelled the emergence of the mobilized colistin-resistance (*mcr*) gene, with enormous implications for the treatment of human infection [7]. Without action, history suggests we will see our remaining effective antibiotics suffer similar fates.

Over-prescription of antibiotics is another critical factor in the evolution of resistance. In some countries, antibiotics are available as over-the-counter medications [9]. By contrast, in countries where antibiotics are prescription-only, the majority of prescriptions are inappropriate [10,11]. For example, physicians may pre-emptively prescribe an antibiotic based on symptoms of disease, without confirmation of a bacterial infection [12]. In other cases, antibiotics are prophylactically prescribed to patients with viral infections, in order to limit the occurrence of a secondary bacterial infection [13]. A push for antimicrobial stewardship has reduced the number of inappropriate antibiotic prescriptions, but vigilance remains important [14].

Bacteria utilize multiple mechanisms in their evolution of antibiotic resistance. Briefly, when a bacterial population is exposed to an antibiotic, the susceptible portion is killed. However, small genetic mutations within a population allow the resistant proportion to recolonize the infection site [15]. Mutations in bacterial populations occur randomly, and relatively slowly. A more rapid form of antibiotic resistance emerges via horizontal gene transfer. Bacteria can acquire genetic material from neighboring resistant-species and incorporate mutant genes into their own genome [16]. Approximately 20% of the *Escherichia coli* genome has been modified over time through horizontal gene transfer [17]. Modifications to bacterial genomes via horizontal gene transfer are commonly seen in biofilm communities, where bacterial communication is essential to their survival [18].

Most commonly, mutations result in the structural modification of an antibiotic target, and subsequent abolishment of antibiotic activity. For example, changes in the structure of the 23S rRNA in *Streptococcus pneumoniae* and *Staphylococcus aureus* confer resistance to the antibiotic linezolid [19]. Resistance to penicillin and other antibiotics in the β-lactam class (cephalosporins, monobactams, carbapenems, and carbacephems) has also emerged owing to the evolution of a diverse range of β-lactamase enzymes. These enzymes directly hydrolyze the antibiotic and render them ineffective [20]. These and various other mechanisms of antibiotic resistance have been extensively reviewed by Munita and Arias [15].

Regulatory and economic hurdles have also stalled the development of new antibiotics by the pharmaceutical industry. As resistant bacteria emerged, and certain antibiotics became ineffective, “me-too” drugs were developed. Minor modifications were made to existing antibiotics; however, many had an identical mechanism of action [21]. Thus, rapid resistance was seen with these compounds. Today, the antibiotic pipeline has been exhausted, with the number of new antibiotics developed seeing a steady decline over the last three decades [2]. Novel drug development is time consuming and costly, thus many pharmaceutical companies have directed their attention towards more profitable drugs. While antibiotic research declines, drug-resistant pathogens are increasing globally [22]. Of the twelve antibiotics released since 2000, eight have widespread resistant clinical isolates. The most recent antibiotic combination, ceftazidime/avibactam, developed in resistant isolates within one year of release to market [23].

## 3. Antimicrobial Peptides in Nature

**Figure 2:**
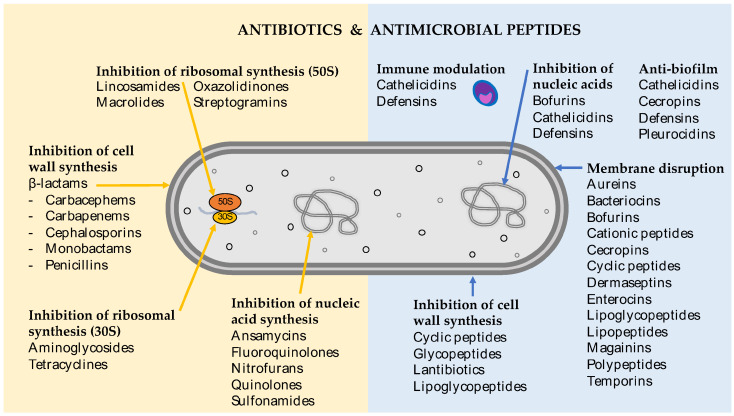
Mechanistic targets of antibiotics and antimicrobial peptides.

There is an undeniable need to research novel antibiotic compounds and new strategies to combat bacterial infections. Antimicrobial peptides and their mimics are rapidly gaining attention as a new class of antimicrobial, with profound clinical potential. Antimicrobial peptides show extraordinary chemical diversity in nature. However, there are some common structural characteristics that set them apart from traditional antibiotics. These peptides usually contain less than 100 amino acids, often including more positively charged residues (such as lysine, arginine, and histidine) and a large portion of hydrophobic residues (>50%) [24]. In addition to their structural differences, antimicrobial peptides often target the bacterial cell membrane directly, as opposed to intracellular machinery (Figure 2).

**Figure 3:**
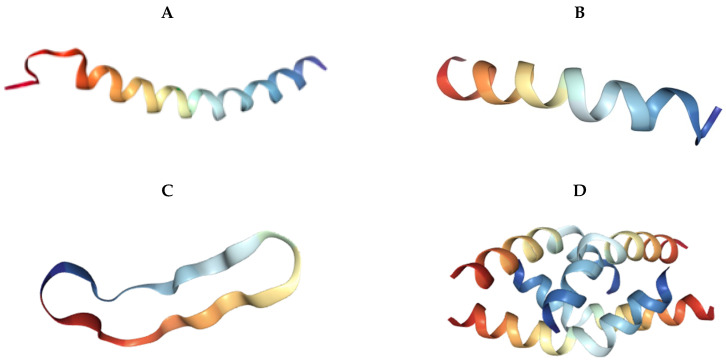

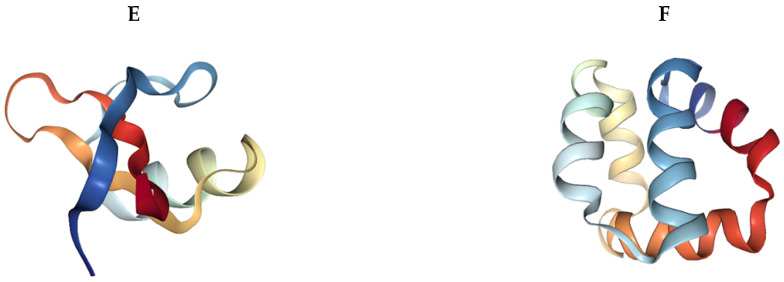
Three-dimensional conformations of natural antimicrobial peptides. Sourced from the Protein Data Bank (https://www.rscb.org). (**A**) LL-37 (Homo sapiens) [26]. (**B**) Magainin-2 (*Xenopus laevis*) [27]. (**C**) RTD-1 (*Rhesus macaque*) [28]. (**D**) Melittin (*Apis mellifera*) [29]. (**E**) TPP3 (Solanum lycopersicum) [30]. (**F**) Bacteriocin AS-48 (Enterococcus faecalis) [31].

Antimicrobial peptides are broadly classified into four different groups based on their structure: α-helical, β-sheet, extended, and cyclic. Some antimicrobial peptides consist entirely of a single helix or sheet, while others have a more complicated structure. The extended peptides are characterized by their lack of recognizable structural motifs. However, they contain high amounts of specific amino acids, such as arginine, tryptophan, glycine, and histidine [25]. The human histatins are particularly rich in histidine residues. The diversity of three-dimensional antimicrobial peptide structures is shown in Figure 3.

Antimicrobial peptides have been identified in all domains of life, where they play an important role in innate immunity. As insects and plants do not have an adaptive immune system, antimicrobial peptides are their primary defense against pathogenic microorganisms [32,33]. Antimicrobial peptides are also produced by bacteria and other microorganisms, where they help microbes to defend their environmental niche [32,33]. Antimicrobial peptides have more diverse roles in higher eukaryotes, including regulation of the innate and adaptive immune pathways [34].

The first evidence for the critical role played by antimicrobial peptides in insect defense systems was obtained in the 1996, when Hoffmann and colleagues demonstrated that removal of the antimicrobial peptide synthesis genetic machinery rendered fruit flies susceptible to fungal infections [35]. Simultaneously, with the discovery of antimicrobial peptides in mammalian skin and demonstration of the importance of antimicrobial peptides in mammalian host defense, scientific and clinical interest in antimicrobial peptides increased [36]. Since then, antimicrobial peptides have been discovered and characterized from almost every multicellular organism. The Antimicrobial Peptide Database (http://aps.unmc.edu/AP/main.php) currently contains more than 3000 antimicrobial peptides, with this number expected to increase in the coming years.

The most prevalent mechanism of action of antimicrobial peptides is via their direct activity on the bacterial cell membrane [37]. The amphipathic nature of antimicrobial peptides contributes to their ability to interact with bacterial membranes. Most antimicrobial peptides have a net positive charge and are thus called cationic antimicrobial peptides. Electrostatic interactions between the cationic antimicrobial peptides and anionic bacterial membranes stabilize the binding of these antimicrobial peptides to the membranes. Subsequently, the bacterial membrane is disrupted, leading to insertion of antimicrobial peptides into the membranes and, often, the formation of pores [33]. Various mechanisms have been suggested for the permeation of antimicrobial peptides through bacterial membranes and have been extensively reviewed [38,39]. In summary, antimicrobial peptide binding leads to a breakdown of membrane potential, an alteration in membrane permeability, and metabolite leakage, ultimately causing bacterial cell death.

In addition to their direct activity as antimicrobials, antimicrobial peptides regulate key immunomodulatory mechanisms in the innate immune system (Figure 2). In higher eukaryotes, a class of antimicrobial peptides, called host defense peptides, modulate immune responses by acting as chemoattractants for leukocytes, enhancing leukocyte/monocyte activity and the expression of proinflammatory cytokines [33]. For example, the human antimicrobial peptide, LL-37, a membrane disrupting peptide, also acts as a chemoattractant for monocytes, neutrophils, mast cells, and T cells [33].

Antimicrobial peptides produced by vertebrates are grouped into two major families: defensins and cathelicidins. LL-37 is the only human cathelicidin peptide. In non-polar environments, LL-37 has an α-helical morphology [33]. Defensins also exhibit chemotactic properties alongside their bactericidal effects. Defensins are produced by multiple cell types, such as neutrophils, macrophages, cardiomyocytes, lymphocytes, keratinocytes, and intestinal epithelial cells [37]. In addition to their role as chemoattractants, defensins are also involved in the activation of classical complement pathways [40].

An advantage of antimicrobial peptides is their action on distinct biological targets to traditional antibiotics [41]. Moreover, a unique quality of many antimicrobial peptides is their multiple mechanisms of action, which together contribute to their overall antimicrobial activity. For example, the human cathelicidin LL-37 demonstrates direct antimicrobial killing, immune modulation, and antibiofilm activity [42]. While most commonly known for its action on the bacterial cell membrane, LL-37 is also able to modulate both pro-inflammatory and anti-inflammatory immune responses [43]. Additionally, LL-37 exerts antibiofilm activity at physiologically relevant concentrations, far below its in vitro minimum inhibitory concentration (MIC) [44,45]. Thus, antimicrobial peptides, such as LL-37, have diverse and dose-dependent mechanisms of action.

As many antimicrobial peptides act on lipid components of the bacterial cell membrane, they often demonstrate broad-spectrum antimicrobial activity [46]. Antimicrobial peptides isolated from nature are effective against bacteria (gram-positive and gram-negative), viruses (enveloped and non-enveloped), yeasts, fungi, molds, and parasites [41,47,48]. While a single antimicrobial peptide may not act against all of these pathogens, owing to their mechanism of action, there may be overlap in microbial activity between different microbes with anionic membranes. Paradoxically, some antimicrobial peptides isolated from natural sources can also display species-specific antimicrobial activity [49]. This may be a consequence of a highly specialized environmental niche, where specific antimicrobial peptides present an evolutionary advantage.

For example, human defensins have demonstrated antimicrobial activity against bacteria (gram-positive and gram-negative), fungi, and viruses including human immunodeficiency virus, influenza A, adenovirus, severe acute respiratory syndrome, human papillomavirus, and herpes simplex virus [24]. Meanwhile, other antimicrobial peptides, such as C16G2, have been specifically designed to target strain-specific mutans, leaving the parent strains unaffected [50]. Although not discussed in this review of antimicrobial activity, some antimicrobial peptides have potent anticancer activity, which is currently a highly anticipated field of research [51].

## 4. Therapeutic Potential of Antimicrobial Peptides

As many antimicrobial peptides act directly on the bacterial membrane, as opposed to intracellular targets, they have similar activity against antibiotic-resistant and antibiotic-sensitive organisms. For example, mutations in penicillin-binding proteins of *Staphylococcus aureus* confer methicillin-resistance. However, as antimicrobial peptides target the cell membrane, there is no overlap in the mechanism of action, and no cross-resistance is observed. Considering that antimicrobial peptides have potent activity against multidrug-resistant organisms, they could be used to treat the increasing number of antibiotic-resistant infections [52].

Additionally, the ability for a single antimicrobial peptide to act via multiple mechanisms, and distinct pathways, not only increases its antimicrobial efficacy, but also decreases the propensity for resistance to occur [53]. A compound that acts via multiple pathways reduces the likelihood that bacteria can acquire multiple mutations simultaneously. Moreover, as many antimicrobial peptides act on evolutionarily conserved components of the cell membrane, bacteria must completely redesign the structure of their cell membranes, requiring multiple mutations over a prolonged period of time [54]. It is common in cancer chemotherapy for multiple drugs, with distinct mechanisms, to be used in combination to limit tumour resistance [55]. However, the use of multiple drugs increases the potential side effects and toxicity of chemotherapy. Therefore, a single antimicrobial peptide drug, with multiple complementary mechanisms, may have the same antimicrobial effect with minimal side effects.

These desirable qualities of antimicrobial peptides lead to another potential application—coadministration with antibiotics. Combinational antimicrobial peptide and antibiotic therapy may reduce or bypass the occurrence of antibiotic resistance [56]. For example, combination therapy with the antimicrobial peptide DP7 eradicated vancomycin and azithromycin resistance in *Staphylococcus aureus, Pseudomonas aeruginosa*, and *Escherichia coli* [57]. Additionally, in vitro synergy has been observed between many antimicrobial peptides and antibiotics [58,59,60]. This demonstrates particular clinical relevance where the toxicity or adverse side effects of a drug may be reduced when used in combination, at lower dosages. Not only do antimicrobial peptides demonstrate synergistic activity with antibiotics, but they may also interact synergistically with components of the immune system [61].

When considering antimicrobials peptides for their clinical use, it is important to consider toxicity to eukaryotic cells. A number of antimicrobial peptides have been shown to be highly nephrotoxic, largely owing to their high therapeutic dose [62]. Even commercially available antibiotics, such as colistin, are only used as a last resort because of their nephrotoxicity [63]. Selective drug delivery methods may reduce the systemic toxicity of antimicrobials peptide therapy and explains why many antimicrobials peptides have been developed as topical applications.

Synthetic mimics of antimicrobials peptides represent a promising class of novel antibiotic. They are rationally designed in the laboratory to retain an antimicrobial pharmacophore, while allowing flexibility in the chemical structure to adjust for desirable properties such as improved activity, reduced cytotoxicity, and proteolysis. Synthetic mimics are used to overcome the difficulties of synthesizing non-canonical amino acids and complex structural motifs [64]. Solid-phase synthesis is used to generate a range of antimicrobial peptides, with the ability to easily modify selective moieties [65]. Advantageously, chemically synthesized peptide mimics are also more financially viable than conventional methods.

There is an enormous array of potential modifications that can be used to generate new antimicrobial peptides. For example, peptoids are a peptide mimic that are resistant to proteolysis, thus extending their half-life for therapeutic use [66]. In this class of peptide mimics, the side chains are appended to the nitrogen atom, rather than the alpha carbons. Synthetic mimics of the antimicrobial peptide magainin have been developed to tune the conformation of aromatic groups and adjust the overall charge on the molecule. One analogue demonstrated activity against two-hundred strains of *Staphylococcus aureus* and *Escherichia coli*, compared with eight for the endogenous magainin [67]. Enhanced immunomodulatory activity was also seen in this magainin mimic, including neutrophil chemoattractant and enhanced macrophage activation.

## 5. Commercially Available Peptide-Based Antibiotics

Currently, there are ten commercially available peptide-based antimicrobials (Table 1). Similar to many of the traditional antibiotics, seven of these active compounds were isolated from bacterial species. The remaining three are semi-synthetic derivates of existing compounds. All of these peptide-based antibiotics act on the bacterial cell membrane, either directly (membrane lysis) or indirectly (inhibition of cell wall synthesis).

While many antimicrobial peptides have similar mechanistic targets (bacterial cell membrane), their chemical composition can be very broad, as seen in the peptide-based antibiotics currently on the market (Table 1). Polypeptides are a chain of peptide-bonded amino acids. Many peptides are linear in aqueous solutions; because of their amphipathic nature, however, they are able to change their three-dimensional conformation upon interaction with the bacterial cell membrane [68]. Compared with their linear counterparts, cyclic peptides have increased stability in vivo [69]. Glycopeptides are a class of antibiotic, often produced by soil bacteria. The three glycopeptide derivatives (dalbavancin, oritavancin, and telavancin) each have a lipid moiety attached to the peptide backbone, increasing their affinity for the bacterial cell membrane [70].

There remains a gap in the market for compounds that are effective against gram-negative bacteria [71]. The World Health Organisation has published a list of “priority pathogens”, or ESKAPE pathogens, listing multidrug-resistant bacteria that pose a great threat to human health [72]. Of the six critical ESKAPE pathogens, four are gram-negative bacteria. Traditionally, these bacteria have been harder to kill, often because of their cell wall composition and the increased number of drug efflux pumps [72]. While colistin and polymyxin B have gram-negative activity, because of their severe toxicity, they are reserved as last-resort treatments when other options have been exhausted. Gramicidin D has some activity against gram-negative bacteria. However, it shows a strong preference against gram-positive cell membranes. Again, because of its cytotoxicity, gramicidin is only used as a topical agent.

Previously, researchers suggested that antimicrobial peptides had a lower propensity for resistance. However, vancomycin-resistant enterococcus is an increasingly relevant nosocomial pathogen. Telavancin and oritavancin are both peptide-based antibiotics that were developed in response to vancomycin-resistant bacteria. Synthetic modifications to the vancomycin structure increased activity and did not confer cross resistance in vancomycin-resistant organisms [73]. Additionally, the mobilized colistin resistance (*mcr*) gene is commonly found in agricultural samples. The implications of this widespread resistance towards colistin have prompted several countries to ban its use outside of the hospital system. These observations highlight the need for antimicrobial stewardship, and act as a warning for future therapeutics.

**Table 1:** Commercially available peptide-based antibiotics.

Active Ingredient	Origin	Target Organism	Class	Mechanism of Action	Indication	Dosage	Route of Administration	Ref
Bacitracin	Bacteria (*Bacillus subtilis*)	Gram-positive bacteria	Cyclic peptide	Inhibits cell wall synthesis	Skin infections	500 units/g 500 units/g 5000 units/vial	Topical Ophthalmic Intramuscular	[74]
Dalbavancin	Teicoplanin derivative	Gram-positive bacteria	Lipoglycopeptide	Inhibits cell wall synthesis	Skin infections	1000 mg/vial	Intravenous	[75,76]
Daptomycin	Bacteria (*Streptomyces roseosporus*)	Gram-positive bacteria	Lipopeptide	Membrane lysis	Skin infections	500 mg/vial	Intravenous	[77]
Colistin	Bacteria (*Bacillus polymyxa*)	Gram-negative bacteria	Cyclic peptide	Membrane lysis	Multi drug-resistant gram-negative infections	150 mg/vial	Intravenous	[78,79]
Gramicidin D	Bacteria (*Bacillus brevis*)	Gram-positive bacteria, some gram-negative bacteria	Mix of three polypeptides	Membrane poration/lysis	Skin and eye infection	0.25 mg/mL 0.025 mg/mL	Topical Ophthalmic	[80]
Oritavancin	Vancomycin derivative	Gram-positive bacteria	Lipoglycopeptide	Membrane lysis and inhibits cell wall synthesis	Skin infections	800 mg/vial	Intravenous	[81]
Polymyxin B	Bacteria (*Bacillus polymyxa*)	Gram-negative bacteria	Polypeptide	Membrane lysis	Urinary tract and bloodstream infections	10,000 units/g 10,000 units/g 500,000 units/vial	Ophthalmic Topical Intravenous	[82]
Teicoplanin	Bacteria (*Actinoplanes teichomyceticus*)	Gram-positive bacteria	Glycopeptide	Inhibits cell wall synthesis	Serious gram-positive infections	400 mg/vial 400 mg/vial	Intramuscular Intravenous	[83]
Telavancin	Vancomycin derivative	Gram-positive bacteria	Lipoglycopeptide	Membrane lysis and inhibits cell wall synthesis	Skin infections	750 mg/vial	Intravenous	[84]
Vancomycin	Bacteria (*Amycolatopsis orientalis*)	Gram-positive bacteria	Glycopeptide	Inhibits cell wall synthesis	Serious gram-positive infections	250 mg 10 g/vial	Oral Intravenous	[85]

As research continues into the factors that determine resistance, the origin of each antimicrobial peptide is proving to be important. Antimicrobial peptides found in nature are classified as either ribosomally synthesized or non-ribosomally synthesized [53]. Non-ribosomally synthesized peptides are produced by bacteria and fungi [53]. These include bacitracin, daptomycin, colistin, gramicidin, polymyxin B, teicoplanin, and vancomycin. They utilize peptide synthetases to catalyze the production of peptides. In contrast, ribosomally synthesized peptides are produced by plants, animals, and some bacteria, and are found as evolutionarily conserved peptides of the innate immune defenses [53]. These include defensins, indolicidin, lactoferricin, magainins, and melittin. It has been proposed that the acquisition of resistance towards ribosomally synthesized peptides is lower, compared with non-ribosomally synthesized peptides [86]. Evidently, there is an unknown biological significance of the origin of each antimicrobial peptide. Thus, pursuing drug derivatives that are based on ribosomally synthesized peptides may reduce clinical resistance. However, more research is needed to understand this trend.

The design and optimization of peptide mimetics represents a promising avenue for new bioactive compounds. For example, telavancin is a semi-synthetic derivative of vancomycin. The hydrophobic (decylaminoethyl) side chain, appended to the vancosamine sugar, aids in the attachment to bacterial cell membranes [87]. An additional hydrophilic (phosphonmethyl aminomethyl) attachment to the resorcinol moiety increases the half-life of the compound [87]. Telavancin has a proposed second mechanism of action of membrane lysis. It is thought that hydrophobic appendage interacts with lipid II, a peptoglycan embedded in the bacterial cell membrane [88]. While the precise molecular mechanisms remain to be elucidated, preliminary studies in *Staphylococcus aureus* demonstrate that lipid II binding rapidly depolarizes the cell membrane [89].

Antimicrobial peptides are often susceptible to proteases in serum, and may display eukaryotic cell toxicity in high therapeutic doses [90]. Consequently, they are often developed as topical antibacterial agents. Bacitracin and gramicidin are exclusively used as topical agents owing to their protease degradation and hemolytic side effects. However, topical applications come with their own unique challenges. For example, topical gels and creams require sufficient tissue penetration in order to be effective against skin wounds [91]. Thus, drug delivery plays an important role in the efficacy of antimicrobials. Therapeutic development of antimicrobial peptides should focus on optimizing each individual compound to suit the chosen delivery method. Synthetic modifications of antimicrobial peptides may reduce the toxicity and/or increase stability and allow for greater drug delivery options.

## 6. Antimicrobial Peptides in Clinical Trials

The vast number of antimicrobial peptides entering clinical trials reflects their therapeutic potential (Table 2). The antimicrobial peptides in clinical development can be categorized into three distinct approaches: (i) direct antimicrobial activity via the cell membrane, (ii) indirect antimicrobial activity via immune modulation, and (iii) inhibition of intracellular functions. Of the forty-four peptides undergoing clinical and pre-clinical trials, thirty-five compounds act directly on the bacterial cell membrane, where eight target the immune system to modulate the body’s response to infection and three act on an intracellular targets (Table 2). Sixteen of these compounds demonstrate broad-spectrum activity, addressing the need for gram-negative activity.

While antimicrobial peptides themselves are a new class of antibiotic, their unique mechanism of action has also opened a plethora of novel applications. For example, the membrane-active, chimeric peptides melimine, and Mel4 retain antimicrobial activity when covalently bound to contact lens surfaces [92,93]. Moreover, the coated lenses were active after repeated microbial exposure [94]. The covalent attachment of an antimicrobial molecule removes the need for recurrent dosing and avoids non-compliance of individual dosing regimens. A phase III clinical trial of covalently attached Mel4 contact lenses found that, with extended wear (14 days), the incidence of corneal infiltration was reduced by 50% [95]. No cytotoxicity or corneal irritation was observed.

**Table 2:** Peptide-based antimicrobial compounds in clinical trials.

Name	Origin	Target Organism	Class	Mechanism of Action	Indication	Dosage	Route of Administration	Ref
Phase 3								
Dusquetide (IMX942, SGX942)	Rational drug design	p62 protein (sequestosome-1)	Synthetic peptide	Immune modulation	Oral complications of chemotherapy	1.5 mg/mL	Intravenous	NCT03237325
Iseganan (IB-367)	Protegrin analogue	Broad spectrum antibacterial	Synthetic peptide	Bacterial membrane disruption	Oral complications of radiation therapy, Ventilator-associated pneumonia	Undefined 9 mg	Oral rinse Inhalation	NCT00022373 NCT00118781
Mel4	Melimine analogue	Broad spectrum antibacterial	Synthetic chimeric peptide	Bacterial membrane disruption	Keratitis	Undefined	Ocular	ACTRN1261500072556
Murepavadin (POL7080)	Protegrin-1 synthetic mimic	*Pseudomonas*	Synthetic peptide	Bacterial membrane disruption via LptD binding	Ventilator-associated pneumonia	Undefined	Intravenous	NCT03409679
Omiganan (MX-226)	Indolicidin analogue	Broad spectrum antifungal, antibacterial	Synthetic peptide	Bacterial membrane disruption	Severe papulopustular rosacea	Undefined	Topical	NCT02576847
p2TA (AB103)	Rational drug design	CD28 receptor on T-helper 1 lymphocytes	Synthetic peptide	Immune modulation	Necrotizing soft tissue infections	0.5 mg/kg	Intravenous	NCT02469857
Pexiganan (MSI-78)	Magainin analogue	Broad spectrum antibacterial	Synthetic peptide	Bacterial membrane disruption	Diabetic foot ulcers	0.8% *w*/*w*	Topical	NCT01590758
Surotomycin (CB-183,315)	Daptomycin analogue	Gram-positive bacteria	Synthetic cyclic lipopeptide	Bacterial membrane disruption	*Clostridium difficile* infection	250 mg	Oral	NCT01597505
Talactoferrin (TLF, rhLF)	Lactoferrin analogue	Gastrointestinal epithelium	Synthetic glycoprotein	Immune modulation	Severe sepsis	100 mg/mL	Oral solution	NCT01273779
Phase 2								
Brilacidin (PMX-30063)	Defensin mimetic	Broad spectrum antibacterial	Arylamide foldamer	Bacterial membrane disruption	Oral complications of radiation therapy	3 mg/mL	Oral rinse	NCT02324335
C16G2	Novispirin analogue	*Streptococcus mutans*	Synthetic peptide	Strain selective membrane disruption, intracellular targets	Dental caries	13.6 mg 9.2 mg	Oral varnish Oral strip	NCT03196219
DPK 060 (GKH17-WWW)	Human protein kininogen derivative	Broad spectrum antibacterial	Synthetic peptide	Bacterial membrane disruption	Acute otitis externa	2% *w*/*w*	Auricular	NCT01447017
EA-230	Human chorionic gonadotrophin hormone derivative	Proinflammatory immune pathway	Synthetic linear tetrapeptide	Immune modulation	Systemic inflammatory response syndrome	90 mg/kg	Intravenous	NCT03145220
Exeporfinium chloride (XF-73)	Dicationic porphyrin derivative	Broad spectrum antibacterial	Synthetic porphyrin	Bacterial membrane disruption	*Staphylococcal* infections	0.2% *w*/*w*	Nasal gel	NCT03915470
LL-37	Human cathelicidin LL-37	Antibacterial, antibiofilm	Cathelicidin	Bacterial membrane disruption, immune modulation	Diabetic foot ulcer	0.5 mg/mL	Topical	NCT04098562
Lytixar (LTX-109)	Host defense peptide derivative	Broad spectrum antibacterial	Synthetic Oligopeptide	Bacterial membrane disruption	Gram-positive skin infections	5% *w*/*w*	Topical	NCT01223222
Melimine	Melittin / protamine splice	Broad spectrum antibacterial	Synthetic chimeric peptide	Bacterial membrane disruption	Keratitis	Undefined	Ocular	ACTRN12613000369729
Novexatin (NP213)	Rational drug design	Antifungal	Synthetic, cyclic peptide	Bacterial membrane disruption	Fungal nail infection	10% *w*/*w*	Topical	NCT02933879
OP-145	Human cathelicidin LL-37 derivative	Broad spectrum antibacterial	Synthetic peptide	Bacterial membrane disruption	Chronic suppurative otitis media	0.5 mg/mL	Auricular	ISRCTN12149720
Opebacan (rBPI21, neuprex)	Human recombinant endotoxin-binding protein	Lipopolysaccharides/endotoxins of gram-negative bacteria	Synthetic peptide	Bacterial membrane disruption	Graft versus host disease	4 mg/kg	Intravenous	NCT00454155
PAC113 (Nal-P-113)	Histatin analogue	Broad spectrum antibacterial	Synthetic peptide	Bacterial membrane disruption, immune modulation, anti-biofilm	Oral candidiasis	0.15%	Oral rinse	NCT00659971
XOMA-629 (XMP-629)	Human recombinant endotoxin-binding protein	Lipopolysaccharides/endotoxins of gram-negative bacteria	Synthetic peptide	Bacterial membrane disruption	Bacterial skin infections	1% *w*/*w*	Topical	[96]
Phase 1								
hLF1-11	Lactoferrin derivative	Broad spectrum antibacterial, antifungal	Synthetic peptide	Chelating agent, immune modulation	*Staphylococcal* bacteremia	0.5 mg	Intravenous	NCT00509847
WLBU2 (PLG0206)	Rationally designed	Broad spectrum antibacterial, antiviral	Synthetic peptide	Bacterial membrane disruption	Microbial infections	3 mg/kg	Intravenous	ACTRN12618001920280
Preclinical								
Bac8c	Bactenecin derivative	Broad spectrum antibacterial	Synthetic peptide	Bacterial membrane disruption	Dental carries	MIC *E. coli* 6 μg/mL	Oral spray	[97,98,99]
Bacteriocin OR-7	*Lactobacillus salivarius*	Gram negative bacteria, *campylobacter jejuni*	Bacteriocin	Bacterial membrane disruption	Bacterial infections	MIC *C. coli* 0.5 μg/mL	Undefined	[100,101]
Buforin II	*Bufo bufo gargarizans*	Broad spectrum antibacterial	Bofurin I	Nucleic acid binding	*A. baumannii* infections *E. coli* infections	1 mg/kg 0.05 mg/mL	Intravenous Oral	[102,103,104,105]
CA(1-7)M(2-9)	Cecropin A/melittin splice	Broad spectrum antibacterial	Synthetic chimeric peptide	Bacterial membrane disruption	Bacterial infections	MIC *A. baumannii* 2 µg/mL	Undefined	[106,107]
Colicin E1	*Escherichia coli H22*	Antibacterial	Bacteriocin	Bacterial membrane disruption	Bacterial infections	MIC *E. coli* 1 µg/mL	Undefined	[108,109]
Demegel (D2A21)	Cecropin analogue	Antifungal, antibacterial	Synthetic peptide	Bacterial, fungal membrane disruption	Burn wounds	1.5% *w*/*w*	Topical	[110,111]
ETD151	Heliomycin analogue	Antifungal	Synthetic peptide	Fungal membrane disruption	Fungal infections	Undefined	Intravenous	[112]
HB-107	Cecropin B	Wound healing	Cecropin B fragment	Undefined, nonbacteriostatic	Wound infections	100 µg/mL	Topical	[113,114]
HB-50	Cecropin analogue	Broad spectrum antibacterial	Synthetic peptide	Bacterial membrane disruption	Wound infections	1%	Topical	[110,115]
HB1345	Rational design	Broad spectrum antibacterial, anti-inflammatory	Synthetic lipohexapeptide	Bacterial membrane disruption	Skin infections, acne	MIC *P. acnes* 1 µg/mL	Topical	[116,117]
IDR-1002	Bactenecin derivative	*Staphylococcus aureus*	Synthetic peptide	Immune modulation	*P. aeruginosa* lung infections	50 µM	Intratracheal	[118,119]
Lactocin 160	*Lactobacillus rhamnosus*	Antibacterial, *Gardnerella vaginalis*	Bacteriocin	Bacterial membrane disruption	Bacterial vaginosis	10 mg/mL	Intravaginal	[120]
Nisin A	*Lactococcus lactis*	Antibacterial, spermicidal contraceptive	Type A lantibiotic, bacteriocin	Bacterial membrane disruption	Bacterial infections	8 µg/mL	Undefined	[121,122]
Novarifyn (NP432)	Rationally designed	Broad spectrum antibacterial	Synthetic peptide	Bacterial membrane disruption	Bacterial infections	Undefined	Topical	[123,124]
Pediocin PA-1	*Pediococcus acidilactici* UL5	Antibacterial	Bacteriocin	Bacterial membrane disruption	Bacterial infections	1.8 nM	Undefined	[125,126]
Planosporicin	*Planomonospora*	Gram-positive bacteria	Lantibiotic	Inhibition of cell wall biosynthesis	Methicillin-resistant *S. aureus* infections	MIC *S. aureus* 2 µg/mL	Undefined	[127,128]
Ruminococcin C	*Ruminococcus gnavus*	Anti-clostridial	Bacteriocin	Bacterial membrane disruption	Gastrointestinal infections	1.2 µM	Oral	[129,130,131]
SB006 (M6)	Rational design	Gram-negative bacteria	Synthetic peptide	Bacterial membrane disruption	Bacterial infections	4 µg/mL	Undefined	[132]
Syphaxin (SPX1-22)	*Leptodactylus syphax*	Broad spectrum antibacterial	Ocellatin-S1	Bacterial membrane disruption	Bacterial infections	MIC *S. aureus* 64 µg/mL	Undefined	[133]
Temporin10a	*Rana ornativentris*	Gram-positive bacteria	Temporin	Bacterial membrane disruption	Bacterial infections	MIC *S. aureus* 2 µM	Undefined	[134]

In addition to their antibacterial activity, many antimicrobial peptides act on the fungal cell membrane [135]. Similarly to antibiotics, most antifungal agents act on intracellular targets [136]. Thus, many of the problems with antibiotics also apply to antifungals. The antimicrobial action on the cell membrane reduces the development of drug-resistant strains. *Candida species* are the most common opportunistic fungal pathogens, with limited therapeutic options and increasing antifungal-resistance [136]. Of the peptide-based compounds in clinical trials (Table 2), omiganan, novexatin, hLF(1-11), demegel, and ETD151 all demonstrated activity against *Candida* species, including drug-resistant strains. These compounds are promising candidates to treat fungal infections that are resistant to available antifungal agents.

While the majority of antimicrobial peptides act directly on the bacterial cell membrane, a select few act on intracellular targets. Buforin II is a broad spectrum antibacterial in preclinical trials. Mechanistic studies proved that buforin II caused bacterial cell death, without membrane lysis, even at 5× MIC [103]. While structurally similar to the membrane-active magainin 2, buforin II accumulates inside the cell and binds nucleic acids, resulting in rapid cell death [103]. Planosporicin is a natural peptide with a similar mechanism of action to β-lactam antibiotics. While its primary action is inhibition of cell wall synthesis, a recent paper reports that planosporin also acts as an extracellular signaling molecule to increase its own production [137]. This feed-forward mechanism was shown to produce biologically effective concentrations of planosporin [137].

Alternatively, some antimicrobial peptides do not have direct antibacterial activity. Paradoxically, these compounds may be the strategy we need to limit bacterial resistance. Any biocidal compound will encounter resistant organisms. However, with no bacterial target to modify, resistance cannot occur to these compounds. Many of the compounds in clinical development to treat sepsis act on pro-inflammatory immune pathways. For example, p2TA is a peptide mimic of the CD28 receptor on T-helper 1 lymphocytes and EA-230 is a human chorionic gonadotropin hormone mimic. The immune modulating peptide, dusquetide, is currently in Phase III clinical trial. This peptide mimic acts on the p62 protein expressed on innate immune cells, in order to increase wound debridement and healing [138]. While these peptides act on different biological targets, each attenuates the pro-inflammatory response to bacterial toxins, and thus decreases the incidence of organ failure.

Antimicrobial peptides, such as Nal-P-113, also have antibiofilm activity [139]. Biofilm infections can be up to 1000× more resistant to antibiotics [140], and there is an unmet clinical need for biofilm-acting compounds. As almost all medical device-related infections are biofilm-related, these compounds may have a profound impact on the way we prevent infection [141]. While the exact mechanism of Nal-P-113 is unknown, it was shown to reduce biofilm formation at low concentrations [139]. Interestingly, this is also seen with the human LL-37 peptide [44]. Furthermore, recent data highlight the ability of antimicrobial peptides to infiltrate an established biofilm [142]. While antimicrobial peptides are promising candidates, biofilms are complex biological communities, and more detailed studies are required to evaluate their full potential.

While the currently approved peptide-based antibiotics are mostly isolated from bacteria (Table 1), many of the antimicrobial peptides currently in clinical trials are chemically synthesized peptide mimetics (Table 2). The ability to modify the chemical structure of antimicrobial peptides allows finer tuning of efficacy, toxicity, and stability. The ability to modify a chemical structure to tune for desired properties will enhance a drug’s ability to proceed through clinical trials. Synthetic mimics of antimicrobial peptides further elucidate the structure–activity relationship of antimicrobial activity. For example, in the cecropin family, CA(1-7)M(2-9), demegel, and HB-50 all disrupt the bacterial cell membrane. However, the cecropin mimic, HB-107, is nonbacteriostatic and is thought to act as an immunomodulator [114]. This highlights how subtle changes to peptide structures confer different mechanisms of action.

While the currently approved peptide-based antibiotics are mostly isolated from bacteria (Table 1), many of the antimicrobial peptides currently in clinical trials are chemically synthesized peptide mimetics (Table 2). The ability to modify the chemical structure of antimicrobial peptides allows finer tuning of efficacy, toxicity, and stability. For example, fine tuning of antimicrobial pharmacophores has increased the stability of peptides and increased their clinical potential. While many antimicrobial peptides have been developed as topical agents, a large number have been developed for internal use (Table 2). Synthetic modifications to reduce systemic toxicity allow peptides to be used internally. For example, selective tuning of the membrane-active region of an AS-48 homologue reduced cytotoxicity towards mammalian cells [143].

The range of peptide structures and origins also highlights the diversity of their clinical potential. Peptide-based agents are not limited to one application, but are being developed for an array of indications (Table 2). Antimicrobial peptide treatments range from simple mouth washes to treatment of severe sepsis. While different delivery methods require different dosages, they are comparable to those required for traditional antibiotics (Table 1 and Table 2). Clinical trials have been cautious of toxicity at these doses, as other peptide-based antibiotics (such as colistin) are toxic in high concentrations.

The successful development of antimicrobial peptides as clinical therapeutics remains a challenge. A large number of antimicrobial peptides have failed throughout the various stages of clinical trials. While many show promising activity in animal models and in vitro, this does not always translate to the multifaceted nature of human disease [144]. One reoccurring reason that antimicrobial peptides fail to progress to market is that they do not show improved activity over currently available antibiotics, for a particular indication. Thus, in the clinical trial design, it is important to consider if there is an overwhelming need for a particular indication. Another concern is the level of toxicity. In the case of iseganan, the clinical trial was stopped when patients in the treatment group showed increased mortality compared with placebo [145].

As compounds proceed through clinical trials, the focus shifts from safe drug dosages to drug efficacy. Thus, the primary reason drugs fail at stage 3 is that they fail to reach clinical endpoints for efficacy. Indeed, omiganan, pexiganan, and surotomycin failed to show a benefit over control treatments [146,147,148]. For iseganan and talactoferrin, the studies were halted because of increased mortality in treatment groups compared with controls [149,150]. Meanwhile, murepavadin failed because of acute renal toxicity in the treatment group [151].

## 7. Future Perspectives

There is a global call for action, for the development of novel antimicrobial compounds, in order to avert the next antimicrobial crisis. The large number of antimicrobial peptides proceeding clinical trials reflects their clinical potential. While antimicrobial peptides represent a promising class of antimicrobial compounds, there is still more work to be done. Many of the antimicrobial peptides in clinical trials failed to progress to market owing to inappropriate trail design, or lack of efficacy. Thus, more research into the interaction between peptide-based antimicrobials and the complex human environment will help to evaluate the true potential of these agents. As antimicrobial peptides represent a powerful tool against drug-resistant pathogens, clinical trials should focus on where there is an unmet clinical need in order to gain momentum.

The ability to chemically modify the structure of antimicrobial peptides allows almost infinite possibility. Identifying a common pharmacophore and desirable modifications will enhance the ability of a compound to proceed through clinical trials. Indeed, many of the compounds in clinical trials have some sort of chemical modification to improve their druggability. The development of sophisticated digital libraries and modelling software will further optimize the development of these compounds.

Finally, we must not repeat the mistakes of our past, and must diligently try to limit the rate of resistance towards novel antimicrobial compounds. While research suggests that antimicrobial peptides have a lower propensity for resistance, this phenomenon is an unavoidable evolutionary consequence. The continued development of varied antimicrobial compounds and mechanisms of antimicrobial action will help to limit the impact of antimicrobial resistance. Moreover, when a new antimicrobial drug is released to market, it will require detailed monitoring and stewardship. Limiting the use of antimicrobials in non-essential cases, or coadministration with antibiotics, will further limit the risk of resistant organisms.

## Abbreviations

*mcr*	Mobilized colistin-resistance
*MIC*	Minimum inhibitory concentration
WHO	World Health Organization
